# Composition, Shell Strength, and Metabolizable Energy of *Mulinia lateralis* and *Ischadium recurvum* as Food for Wintering Surf Scoters (*Melanitta perspicillata*)

**DOI:** 10.1371/journal.pone.0119839

**Published:** 2015-05-15

**Authors:** Alicia M. Wells-Berlin, Matthew C. Perry, Richard A. Kohn, Kennedy T. Paynter, Mary Ann Ottinger

**Affiliations:** 1 USGS Patuxent Wildlife Research Center, 12100 Beech Forest Rd., Laurel, Maryland, 20708, United States of America; 2 Department of Animal and Avian Sciences, University of Maryland, 4150 Animal Sciences Bldg, College Park, Maryland, 20742–2311, United States of America; 3 Marine & Estuarine-Environmental Science Program, University of Maryland, 0105 Cole Student Activities Bldg, College Park, Maryland, 20742–5511, United States of America; Phillip Island Nature Parks, AUSTRALIA

## Abstract

Decline in surf scoter (*Melanitta perspicillata*) waterfowl populations wintering in the Chesapeake Bay has been associated with changes in the availability of benthic bivalves. The Bay has become more eutrophic, causing changes in the benthos available to surf scoters. The subsequent decline in oyster beds (*Crassostrea virginica*) has reduced the hard substrate needed by the hooked mussel (*Ischadium recurvum*), one of the primary prey items for surf scoters, causing the surf scoter to switch to a more opportune species, the dwarf surfclam (*Mulinia lateralis*). The composition (macronutrients, minerals, and amino acids), shell strength (N), and metabolizable energy (kJ) of these prey items were quantified to determine the relative foraging values for wintering scoters. Pooled samples of each prey item were analyzed to determine composition. Shell strength (N) was measured using a shell crack compression test. Total collection digestibility trials were conducted on eight captive surf scoters. For the prey size range commonly consumed by surf scoters (6–12 mm for *M*. *lateralis* and 18–24 mm for *I*. *recurvum*), *I*. *recurvum* contained higher ash, protein, lipid, and energy per individual organism than *M*. *lateralis*. *I*. *recurvum* required significantly greater force to crack the shell relative to *M*. *lateralis*. No difference in metabolized energy was observed for these prey items in wintering surf scoters, despite *I*. *recurvum*’s higher ash content and harder shell than *M*. *lateralis*. Therefore, wintering surf scoters were able to obtain the same amount of energy from each prey item, implying that they can sustain themselves if forced to switch prey.

## Introduction

Reports of the Atlantic coast surf scoter populations have shown a steady decline (unpub. USFWS survey data) over the last decade. The primary wintering area for surf scoters along the Atlantic flyway is the Chesapeake Bay. While wintering in the Bay the ducks primarily prey on two food items, the hooked mussel (*Ischadium recurvum*) and dwarf surfclam (*Mulinia lateralis*)[1]. *I*. *recurvum* is an epifaunal organism that utilizes the oyster beds (*Crassostrea virginica*) in the Bay as its primary habitat. However, disease and over harvesting have caused major declines in the oyster populations [2], [3]. With the lack of regeneration of oyster beds and increasing coastal development, there has been an increase in sedimentation rate and reduction in hard substrate, which has negatively impacted other organisms, including *I*. *recurvum*. In addition to a loss in available substrate, there has been an increase in the severity, duration, and frequency of anoxic events in the deep areas of the Bay and some of its tributaries [4], [5], [6]. This has devastating consequences for benthic organisms including reduced benthic biomass, species diversity, and altered structure [7], [5], [8], [9], [10]. With the loss of substrate and the increase in anoxic events, the availability of food for surf scoters has declined, resulting in a decline in the abundance or occurrence of surf scoters.

Benthic communities that have undergone restructuring due to hypoxia [11] tend to be composed of large numbers of opportunistic species and a decreased number of equilibrium (larger and long-lived) species [8]. Opportunistic species are characterized by short life cycles [8], high fecundity, and large recruitment pulses [12]. The prevalence of opportunistic species (e.g. *Mulinia sp*.), and the relatively low percentage of equilibrium species (e.g., *Mya arenaria* and *Macoma balthica*) in the diet of surf scoters, suggests the possibility that hypoxic events may be affecting prey availability and resultant prey selection by scoters [13]. This may be occurring by two different mechanisms. First, the ducks may be induced to feed on another food item, *M*. *lateralis*, which may not be as energetically efficient for them. *M*. *lateralis* may not be available in the same sizes as *I*. *recurvum* and, therefore, may not provide as much energy per dive as hooked mussels. Second, the reduced habitat availability (loss of oyster reefs) has changed the distribution of hooked mussels. This distribution change could result in an increased density in existing habitats, which could benefit scoters; or could lead to overcrowding (increased competition among mussels for space, oxygen, and food; [14]), which would make them an unprofitable food choice for a wintering scoter. There is a minimal density of an organism at which it may no longer be profitable for a scoter to seek it [15]. As the quality and/or quantity of food declines, ducks may travel farther between suitable food items or food patches to maintain adequate energy intake. Therefore, the net energy gain obtained from hooked mussels may be exceeded by the cost associated with obtaining that prey item.

Ultimately, surf scoters should select the prey item that provides the maximum net rate of energy return (energy provided by prey minus energy required to find and consume prey; [16]). Measurements of metabolizable energy are needed to define the efficiency of utilization of nutrients within food, and to classify the nutritional quality of a food item. For a given prey item, the metabolizability of energy is determined by the prey’s chemical makeup and by the digestive physiology of the duck. A duck, which consumes its prey whole, must choose a foraging strategy that compromises efficiency for low digesta volumes and high total rates of nutrient extraction [17].

The goal of this study was to evaluate the composition (macronutrients, minerals, and amino acids), shell strength (N), and metabolizable energy (kJ) of the top two prey items for surf scoters, *I*. *recurvum* and *M*. *lateralis*, to determine whether one prey item was more beneficial than the other. In concurrent experiments, this allowed an estimation of whether the surf scoter could maintain its energetic needs if the availability of either of these two prey items was altered.

## Materials and Methods

### Prey Collection

Both prey items were collected in mesohaline portion of the Chesapeake Bay, primarily in Choptank River and near Poplar Island. *I*. *recurvum* was collected by local oysterman who permitted us to remove mussels from oysters collected using tongs. *M*.*lateralis* was collected using a ponar benthic grab. Large numbers of *M*. *lateralis* were not located so additional quantities, primarily for metabolizable energy trials, were purchased from Woods Hole Marine Laboratory.

### Dry mass, ash mass, and organic matter mass

The dry mass (g), ash mass (g), and organic matter (g; OM) of 30 *I*. *recurvum* and 25 *M*. *lateralis* were measured for each size (length) class (6–12, 12–18, 18–24, 24–30, 30–36, 36–42 mm; Table 1). *M*. *lateralis* samples were not larger than 18 mm so the analyses for this species included only two size classes (6–12, 12–18). To examine the seasonal differences in the dry mass (g), ash mass (g), and OM (g) of *I*. *recurvum*, these analyses were completed on 30 individuals per size class collected throughout the year (January, February, March, May, June, and July). To determine dry mass, all specimens were weighed to the nearest 0.001g and oven dried separately at 50°C to constant mass. Individual whole bivalves were then burned in a muffle furnace at 500°C for 6 hr to yield OM.

**Table 1:** The mean amounts (± 1 SD) of crude protein (g DM/individual), lipid (g DM/individual), and gross energy (kJ/individual) found for each size class (6–12, 12–18, 18–24, 24–30, 30–36, 36–42 mm) of *Ischadium recurvum* collected from the Chesapeake Bay in January and May 2007.

Size Class (mm)	n	Crude Protein (g/ind)	Lipid (g/ind)	Gross Energy (kJ/ind)
**January (Winter)**				
** 6–12**	30	0.007 ± 0.003a	0.004 ± 0.0002	NA
** 12–18**	30	0.018 ± 0.006a	0.002 ± 0.0008a	0.468 ± 0.156a
** 18–24**	30	0.046 ± 0.009a	0.004 ± 0.0009a	1.878 ± 0.370a
** 24–30**	30	0.083 ± 0.016a	0.015 ± 0.003a	2.814 ± 0.532a
** 30–36**	30	0.138 ± 0.030a	0.010 ± 0.002a	4.990 ± 1.086a
** 36–42**	60	0.208 ± 0.053a	0.020 ± 0.005a	6.422 ± 1.623a
**May (Spring)**				
** 6–12**	27	0.005 ± 0.003b	NA	0.097 ± 0.053
** 12–18**	29	0.027 ± 0.009b	0.003 ± 0.001a	0.744 ± 0.240b
** 18–24**	19	0.061 ± 0.014b	0.005 ± 0.001a	2.205 ± 0.487b
** 24–30**	30	0.114 ± 0.024b	0.011 ± 0.002b	4.612 ± 0.971b
** 30–36**	30	0.171 ± 0.033b	0.018 ± 0.003b	7.546 ± 1.459b
** 36–42**	30	0.562 ± 0.136b	0.026 ± 0.006b	11.37 ± 2.757b

Values followed by the same letter were not significantly different (p < 0.05).

### Nutrient Content

To determine nutrient content, ten pooled bivalves of each size class (20–25 individuals for smallest size classes) collected in winter were sent to University of Arkansas’ Center of Excellence for Poultry Science (CEPS) Laboratory. For *I*. *recurvum*, additional pooled samples collected in May were analyzed. Crude protein (% g DM; AOAC 990.03)[18], lipid (% g DM; AOAC 920.39c)[18], gross energy (kJ/g DM; ANSI/ASTM D2015-77)[18], ash (% g DM; AOAC 923.03)[18], dry matter (DM; % g; AOAC 934.01)[18], and mineral content (ppm; AOAC 968.08)[18] of these pooled individuals were determined. Crude protein was determined by freeing nitrogen by combustion at high temperature in pure oxygen, measuring by thermal conductivity, and converting to equivalent protein using an appropriate conversion factor (total N x 6.25). For lipid, a sample was dried with anhydrous ether in a thimble with porosity permitting the passage of ether. Extraction period varied from 4 h at condensation rate of 5–6 drops/s to 16 h at 2–3 drops/s. The extract was dried for 30 minutes at 100°C, cooled, and weighed. The standard test method for gross calorific value of solid fuel by the adiabatic bomb calorimeter was used to determine gross energy amounts. For ash, a 3–5 g sample was placed into and ashing dish that was ignited, cooled in dessicator, and weighed soon after reaching room temperature. The sample was ignited in a furnace at 550°C until light gray ash results, cooled in a desiccator, and weighed soon after reaching room temperature. For dry matter determination, a 2 g sample was dried to constant weight at 95°–100°C under pressure 100 mm Hg. Loss on drying (LOD) was used as an estimate of moisture content. Mineral content was determined by the atomic absorption spectrophotometric method.

These fractions of crude protein, lipid, and gross energy for pooled samples were then converted to absolute amounts (g per individual organism) by multiplying them by the dry mass of each species, size class, and month (January and May) as determined earlier. In addition, to detect any differences in the protein structure of these species, amino acid analyses were completed on these pooled samples at CEPS (AOAC 982.30a)[18]. Acid hydrolysis method for determining amino acid profiles was as follows: a 0.1 g sample was placed in a hydrolysis tube and mixed with 10 mL 6M HCl, frozen in dry ice–alcohol bath, and held under a vacuum of 50 mm for 1 min to seal the tube. The sample was hydrolyzed for 24 h at 110° ± 1°C, cooled, and the hydrolysate was filtered (Whatman No. 1 paper; rinse); rinsed 3 times with H_2_O and filtered with a paper filter each rinse. The filtrate was dried at 65°C under vacuum and then dissolved in buffer for amino acid analysis. All amino acids were analyzed except methionine, cystine and/or cysteine, and tryptophan. The % gram amounts for each amino acid were normalized to a standard (lysine) to better align differences between profiles of each prey item. The mineral and amino acid analyses were conducted on only one pooled sample per size class. It was determined that there was equal contribution by individuals to these data, and with the accuracy of 1–2% for these methodologies, single samples were considered representative of the content for these prey species. Therefore, multiple measurements were not performed by CEPS.

### Shell Strength

Shell strength (N) was measured on 20 individuals of each prey species using a compression test at University of Maryland (UM). An Imada Force Measurement System was used with a digital force gauge, which monitored the force (lb f) applied to the shell surface and recorded the force when the shell cracked. The pressing surfaces of the meter contacted the opposing shells just below the umbo and the force was gradually increased until the shell cracked.

### Ethics Statement

The protocols for the following section entitled “Apparent Metabolizable Energy” were approved by the Patuxent Wildlife Research Center (PWRC) Animal Care and Use Committee and University of Maryland Animal Care and Use Committee (# R-03-06). This study was carried out in strict accordance with the recommendations in the Guide for the Care and Use of Laboratory Animals of the National Institutes of Health.

### Apparent Metabolizable Energy

Nine surf scoters (5 M: 4 F) were raised from eggs collected in 2002 from Lac Malbaie, Quebec, Canada. One female was removed from experiment due to poor health and all samples collected from her were removed from analyses. When not in feeding trials, the scoters were kept in outdoor pens and fed *ad libitum* Mazuri Seaduck Diet (number 5681, PMI Nutrition International Brentwood, MO; 21.5% protein). Grit was provided *ad libitum* next to the feed trays. Grit was not supplied for two weeks before or during the trials to prevent variability due to grit in the excreta mass and nutrient analyses.

*I*. *recurvum* and *M*. *lateralis* were frozen and thawed to room temperature before experiments. The nine surf scoters were randomly placed in individual stainless steel cages with removable trays lined in plastic in May 2007. Each duck was weighed before and after each trial to determine body mass (g) loss. Feeding trials consisted of a 24 hour acclimation period where excreta were collected every 4 hours, a single force feeding of 25 g whole wet mass of clams or mussels, and a 48 hour collection period where excreta were collected every 4 hours. Ducks were force fed 25 grams (whole wet mass) of 12–18 mm *M*. *lateralis* and 25 grams (whole wet mass) of 18–24 mm for *I*. *recurvum*, the two size classes that are commonly consumed by scoters, in a cross-over experimental design. Each scoter was fed the randomly assigned test diet by placing thawed bivalves at the back of the throat with a feline pill gun and flushed down the esophagus with water. Any prey regurgitated immediately was once again force fed and flushed with more water. Any diet regurgitated overnight was weighed and deducted from the amount fed.

The amounts fed (whole wet mass; g; ingesta) were then converted to dry matter (g DM), ash (g DM), lipid (g DM), and gross energy (kJ) using values determined earlier in the study. Nitrogen (g) in the ingesta was determined by multiplying the amount fed (g) on a dry matter basis of protein (g DM) and dividing it by 6.25 [19]. The excreta were collected into plastic urine cups with a spatula, preserved in 10 ml of 0.1 M sulfuric acid, and frozen until analyzed. During analyses, samples were freeze dried and sub samples were ground and homogenized by day for each duck. These homogenized samples were analyzed by CEPS for gross energy (kJ/g DM), lipid (% g DM), nitrogen (% g DM), and ash (% g DM) content. The excreta mass per day (g/day) were multiplied by the gram DM for ash, lipid, and nitrogen to determine absolute amounts of each nutrient for each duck. In addition, the energy in the excreta (kJ/g) was multiplied by the amount of excreta on a dry matter basis per day per duck. Based on these data the following equations were calculated:

Apparent Digestible Energy (ADE; %) = [*(Gross energy intake—gross energy excreted)/Gross energy intake*] * *100%*

Nitrogen Energy Balance (NEB; kJ) = *Nitrogen intake—(Nitrogen excreted x 36*.*5)*; the 36.5 is the mean energy content (kJ) per gram urine-nitrogen in birds [20], [21], [22], [23].

Apparent Metabolizable Energy (AME; %) = [*[Gross energy intake—(gross energy excreted + nitrogen balance) / Gross energy intake]*] * *100%*

The correction for nitrogen balance was needed because the energy in excreta from endogenous sources can otherwise result in underestimates of metabolizable energy [24], [22], [23].

### Analyses

Regression analysis was used to predict changes in dry mass, ash mass, and OM, and shell strength as a function of size class for each prey species. Based on residual plots, the data were log transformed before analyses when it was appropriate. Analysis of variance (ANOVA) and Bonferroni pairwise comparisons were used to detect differences for dry mass, ash mass, organic matter, macronutrient content and shell strength within each size class by season and by species. Two-tailed t-tests were used to detect differences between the two prey items for the metabolizable energy trials. When a significant difference was detected for the dry matter of ingesta (g) the remaining ingesta factors were weighted for dry matter and analysis of variance was used to test for significance. Due to the possible carry over of nutrients from the commercial diet provided during the acclimation period the results obtained on the day the ducks were force fed were excluded. All tests were considered significant at the 5% level and all analyses were completed using SAS (Proc Mixed, [25]).

## Results

### Dry mass, ash mass, and ash free dry mass

**Fig 1:**
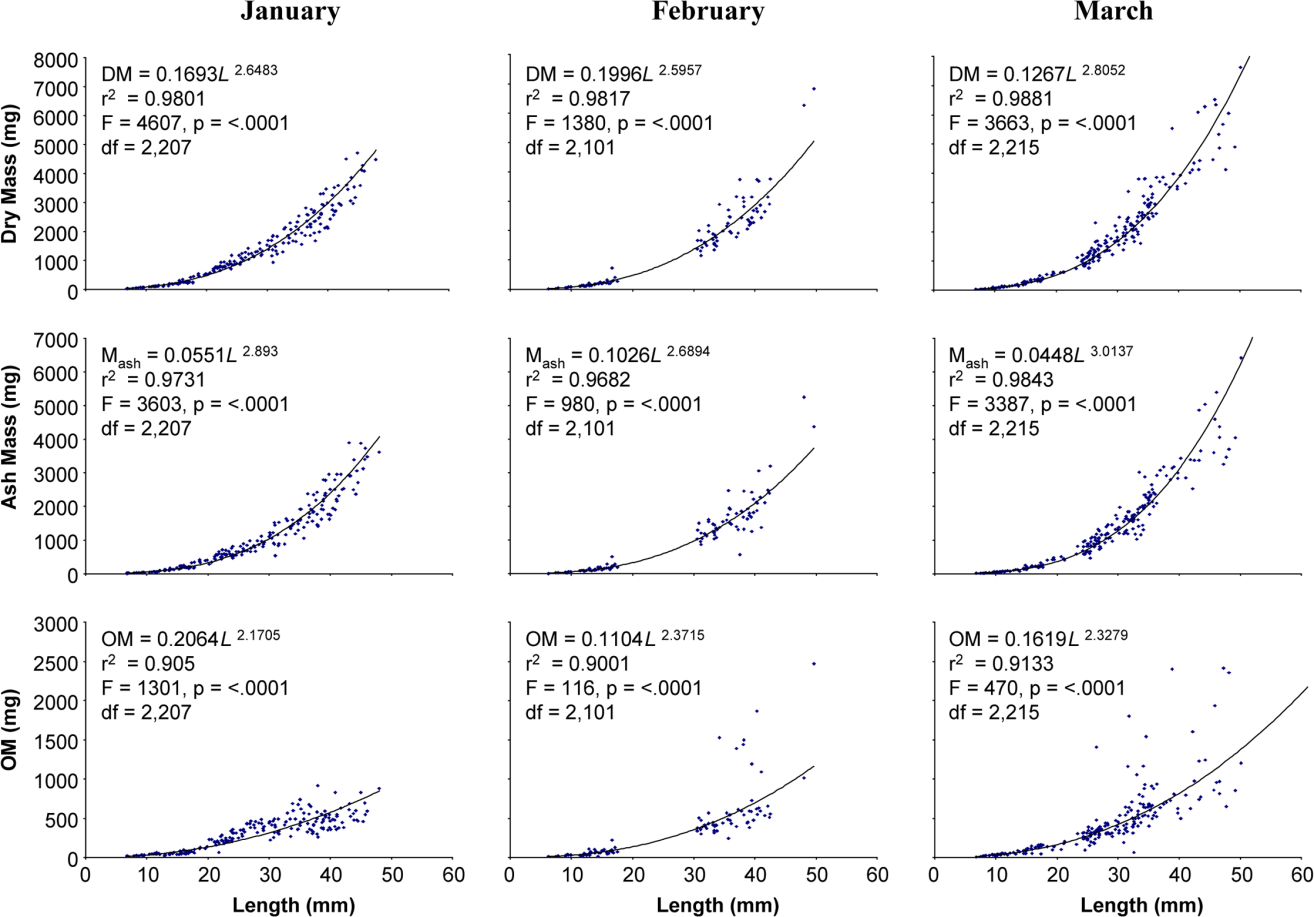
Dry, ash, and organic matter masses of *Ischadium recurvum* collected in winter relative to shell length. Dry mass (DM; mg), ash mass (M_ash_; mg), and organic matter (OM; mg) of *Ischadium recurvum* (including shell) as a function of length (mm) collected from the Chesapeake Bay in January, February, and March 2007. All regressions were significant at the 5% level (p<0.0001).

**Fig 2:**
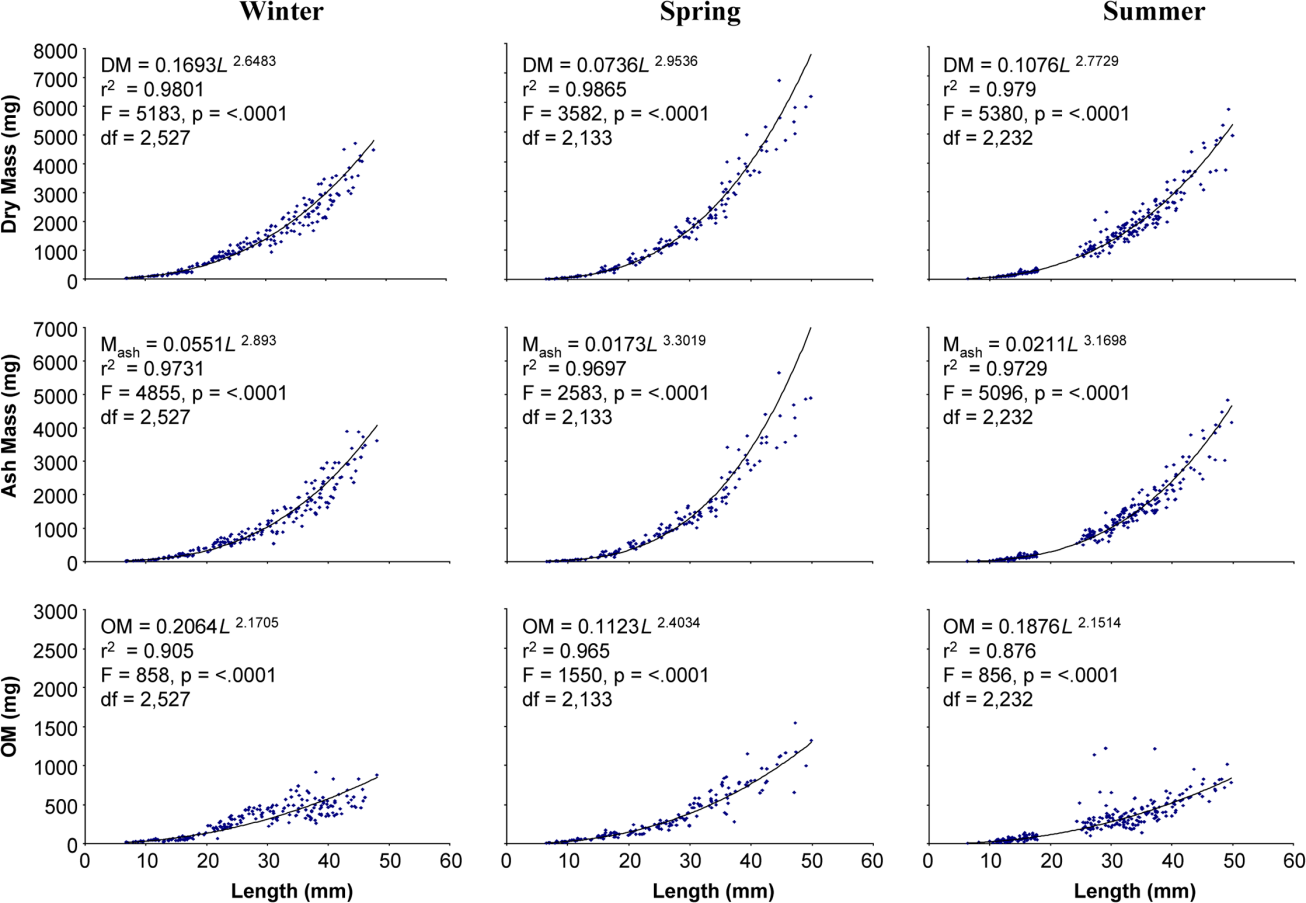
Dry, ash, and organic matter masses of *Ischadium recurvum* collected seasonally relative to shell length. Dry mass (DM; mg), ash mass (M_ash_; mg), and organic matter (OM; mg) of *Ischadium recurvum* (including shell) as a function of length (mm) collected from the Chesapeake Bay in winter, spring, and summer. All regressions were significant at the 5% level (p<0.0001).

**Fig 3:**
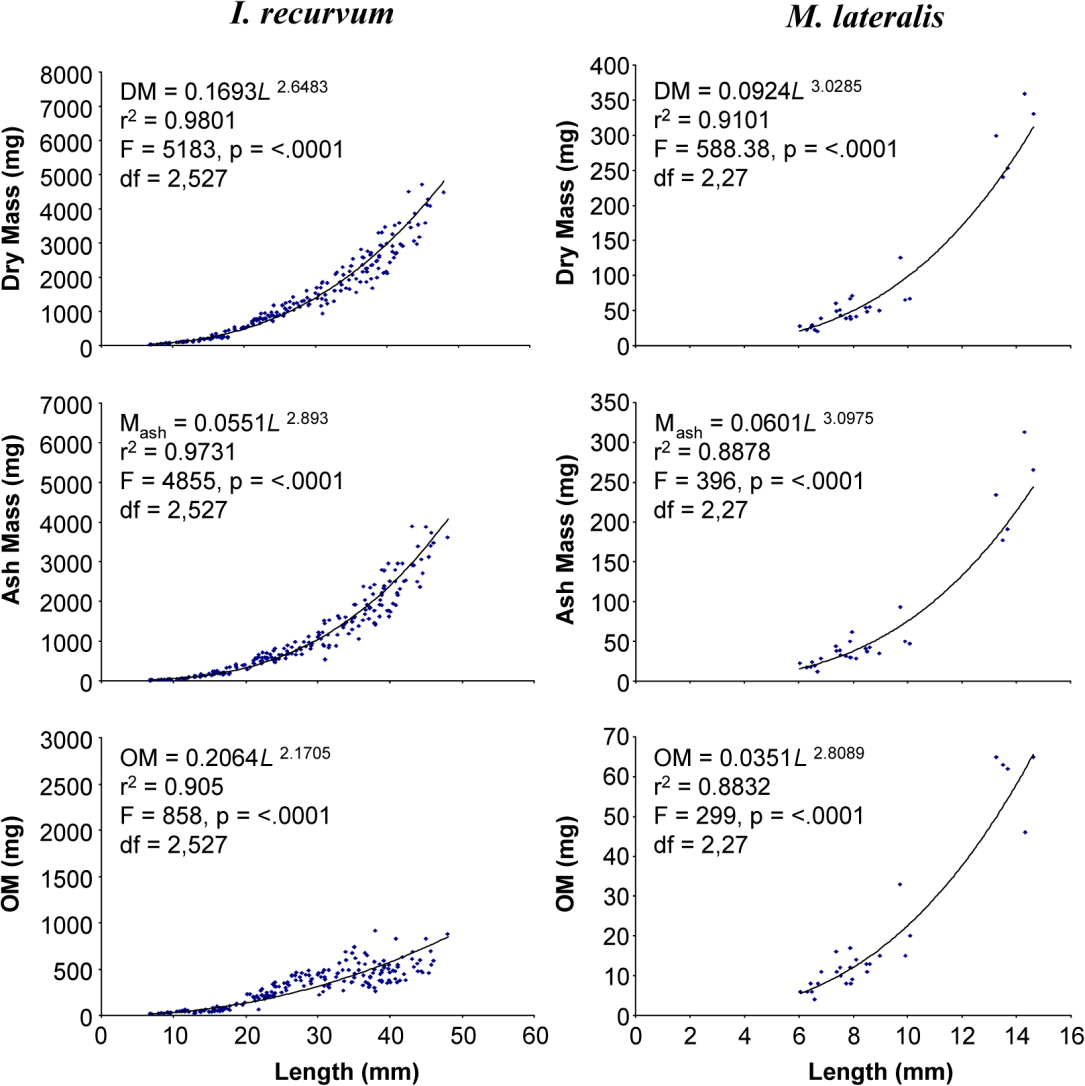
Dry, ash, and organic matter masses of *Ischadium recurvum* and *Mulinia lateralis* relative to shell length. Dry mass (DM; mg), ash mass (M_ash_; mg), and organic matter (OM; mg) of *Ischadium recurvum* and *Mulinia lateralis* (including shell) as a function of length (mm). All regressions were significant at the 5% level (p < 0.0001).

Dry mass, ash mass, and organic matter significantly increased nonlinearly with increasing prey length for all seasons and both prey items (p <0.0001; Figs 1, 2 and 3). Mean comparison on seasonal differences by size classes for *I*. *recurvum* indicated that there was a significant difference in OM between January, March, and May (spring) mussels 18–24 mm in length (p = 0.0023). January mussels of this size contained more OM than March or spring mussels. There was also a significant difference in dry mass and OM between the two prey species within the 6–12 mm size class (p < 0.0001 for both). But in the 12–18 mm size class, there was no significant difference found between the two prey species for dry mass, ash mass, and OM (p = 0.3255, p = 0.0606, and p = 0.3239, respectively).

### Nutrient Content

#### Macronutrients

In general, crude protein, ash, lipid, and gross energy all increased with increasing length for *I*. *recurvum* regardless of season (Table 1). Crude protein (g per individual) contained in spring mussels was significantly higher for all size classes except for the smallest mussels where crude protein content was significantly lower. Spring lipid content (g per individual) was significantly higher than winter for all the larger size classes, except for 30–36 mm where lipid content in winter was higher than in spring. The lipid content in the smaller size classes was not significantly different between seasons. The gross energy content in spring mussels was significantly higher for all size classes than winter mussels. When *M*. *lateralis* and *I*. *recurvum* were compared within the same size class (6–18mm) and the same season (winter), *M*. *lateralis* contained significantly less crude protein and gross energy, but similar amounts of lipid as *I*. *recurvum* (Table 2). When these two prey items were compared based on the size classes commonly consumed by surf scoters, *M*. *lateralis* contained significantly less crude protein, lipid, and gross energy than *I*. *recurvum*.

**Table 2:** The mean amounts (± 1 SD) of crude protein (g DM/individual), lipid (g DM/individual), and gross energy (kJ/individual) found for *Ischadium recurvum* and *Mulinia lateralis*, the top two prey items consumed by wintering surf scoters in the Chesapeake Bay.

Size Class (mm)	n	Crude Protein (g/ind.)	Lipid (g/ind.)	Gross Energy (kJ/ind.)
***I*. *recurvum***				
** 6–18**	60	0.012 ± 0.008b	0.001 ± 0.001b	0.468 ± 0.156b
** 18–24**	30	0.046 ± 0.009a	0.004 ± 0.0009a	1.878 ± 0.370a
***M*. *lateralis***				
** 6–18**	29	0.003 ± 0.003c	0.001 ± 0.001b	0.015 ± 0.016c

Values followed by the same letter were not significantly different (p < 0.05).

#### Minerals

*M*. *lateralis* contained 82.6% more potassium, 95.6% more calcium, 94.7% more selenium, and 80.2% more sodium than *I*. *recurvum* (Table 3). *I*. *recurvum* contained 59.8% more phosphorus, 85.1% more magnesium, 19.6% more iron, 13.0% more manganese, 61.7% more zinc, and 5.0% more copper than *M*. *lateralis*. *M*. *lateralis* contained no aluminum unlike *I*. *recurvum*, which contained 294 ppm of aluminum.

**Table 3:** The mineral content (ppm) determined for 6–18 mm *Ischadium recurvum* and *Mulinia lateralis* collected in winter from the Chesapeake Bay and for *I*. *recurvum* collected from the Bay in winter and spring by size class (6–12, 12–18, 18–24, 24–30, 30–36, 36–42 mm.)

Species	Season	Size	P	K	Ca	Mg	S	Na	Fe	Mn	Zn	Cu	Al
			ppm										
*Mulinia lateralis*	Winter	6–18	540	2436	348413	598	7382	6240	103	18.1	11.9	0.94	0
*Ischadium recurvum*	Winter	6–18	903	2011	333198	703	6987	5006	526	140	19	19	294
*Ischadium recurvum*	Winter	6–12	916	1906	335136	703	7038	5069	511	126	19.5	21.3	286
*Ischadium recurvum*	Spring	6–12	750	1982	328735	547	6735	5000	291	86.0	14.2	8.42	175
*Ischadium recurvum*	Winter	12–18	890	2116	331260	703	6937	4942	540	154	19.1	16.1	301
*Ischadium recurvum*	Spring	12–18	1095	2897	336866	603	7150	5238	287	59.4	13.8	6.10	167
*Ischadium recurvum*	Winter	18–24	1162	2696	331900	977	7550	5538	997	209	22.4	9.12	588
*Ischadium recurvum*	Spring	18–24	1134	3160	327615	678	7087	5001	312	57.8	13.0	5.06	185
*Ischadium recurvum*	Winter	24–30	963	2515	312664	930	6970	5318	1191	285	19.2	9.53	713
*Ischadium recurvum*	Spring	24–30	1610	4541	293973	899	6904	5545	640	67.8	16.3	7.25	415
*Ischadium recurvum*	Winter	30–36	1112	3370	282631	1073	6520	5727	1022	310	28.8	28.6	601
*Ischadium recurvum*	Spring	30–36	1230	3803	315862	923	7081	5493	499	79.1	12.9	5.51	322
*Ischadium recurvum*	Winter	36–42	976	3050	300079	1037	6806	5949	938	350	24.7	17.7	544
*Ischadium recurvum*	Spring	36–42	1055	3573	321770	1077	7207	5932	650	91.8	19.8	5.59	421

When a seasonal comparison was made by size class on *I*. *recurvum*, winter mussels consisted of more iron, manganese, zinc, copper, and aluminum than spring mussels for all size classes (Table 3). However, spring mussels contained more potassium and phosphorus than winter mussels. For most of the size classes, winter mussels predominantly contained more magnesium than spring mussels. There did not appear to be any seasonal differences in calcium and selenium and no apparent influence of size (length) of the mussel on its mineral content was detected.

#### Amino Acids

**Fig 4:**
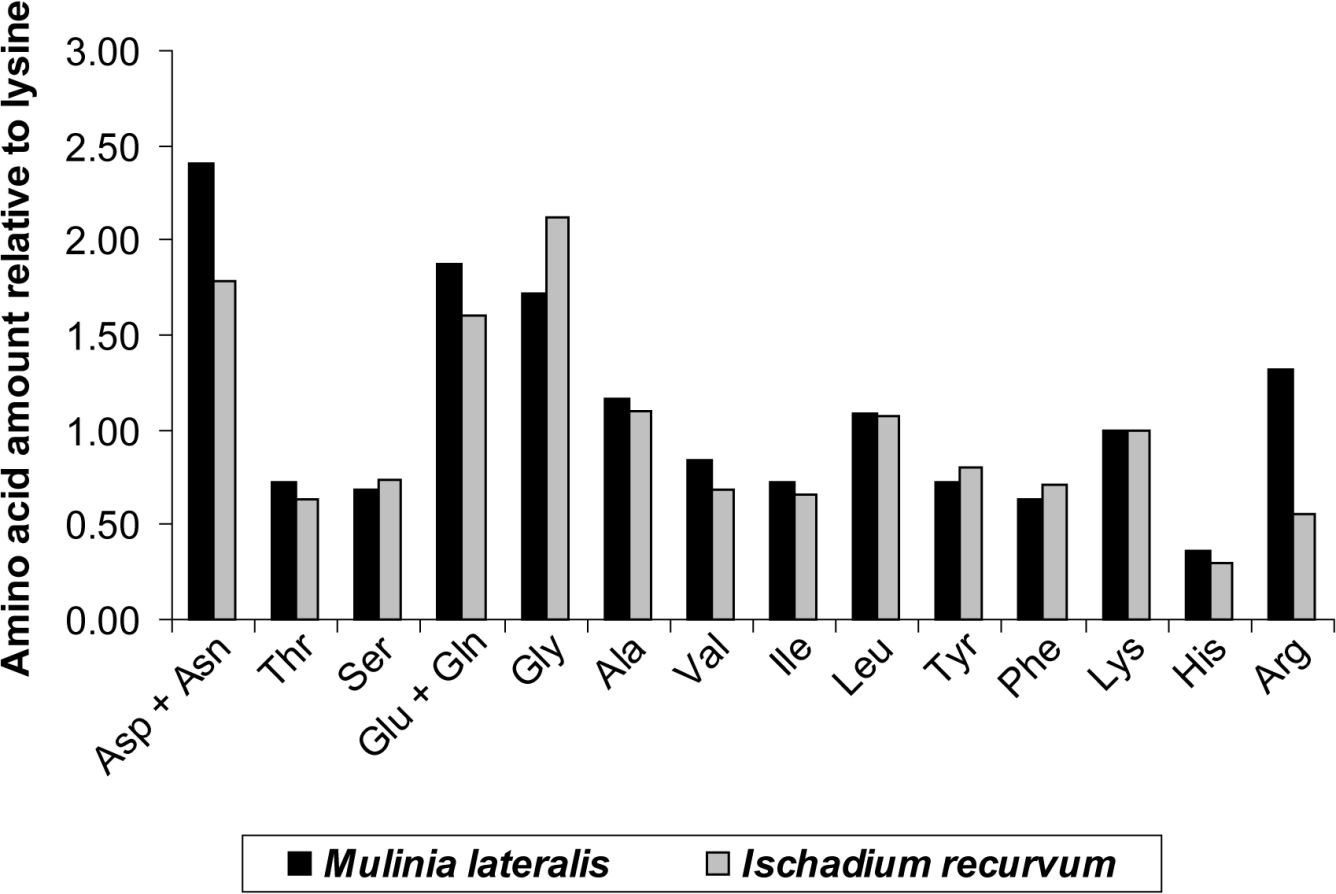
Amino acid amounts relative to lysine for *Mulinia lateralis* and *Ischadium recurvum* collected in winter. The amino acid amounts relative to lysine for *Mulinia lateralis* and *Ischadium recurvum* collected in winter from the Chesapeake Bay. Asp = Aspartic acid; Asn = Asparagine; Thr = Threonine; Ser = Serine; Glu = Glutamic acid; Gln = Glutamine; Gly = Glycine; Ala = Alanine; Val = Valine; Ile = Isoleucine; Leu = Leucine; Tyr = Tyrosine; Phe = Phenylalanine; Lys = Lysine; His = Histidine; and Arg = Arginine.

**Fig 5:**
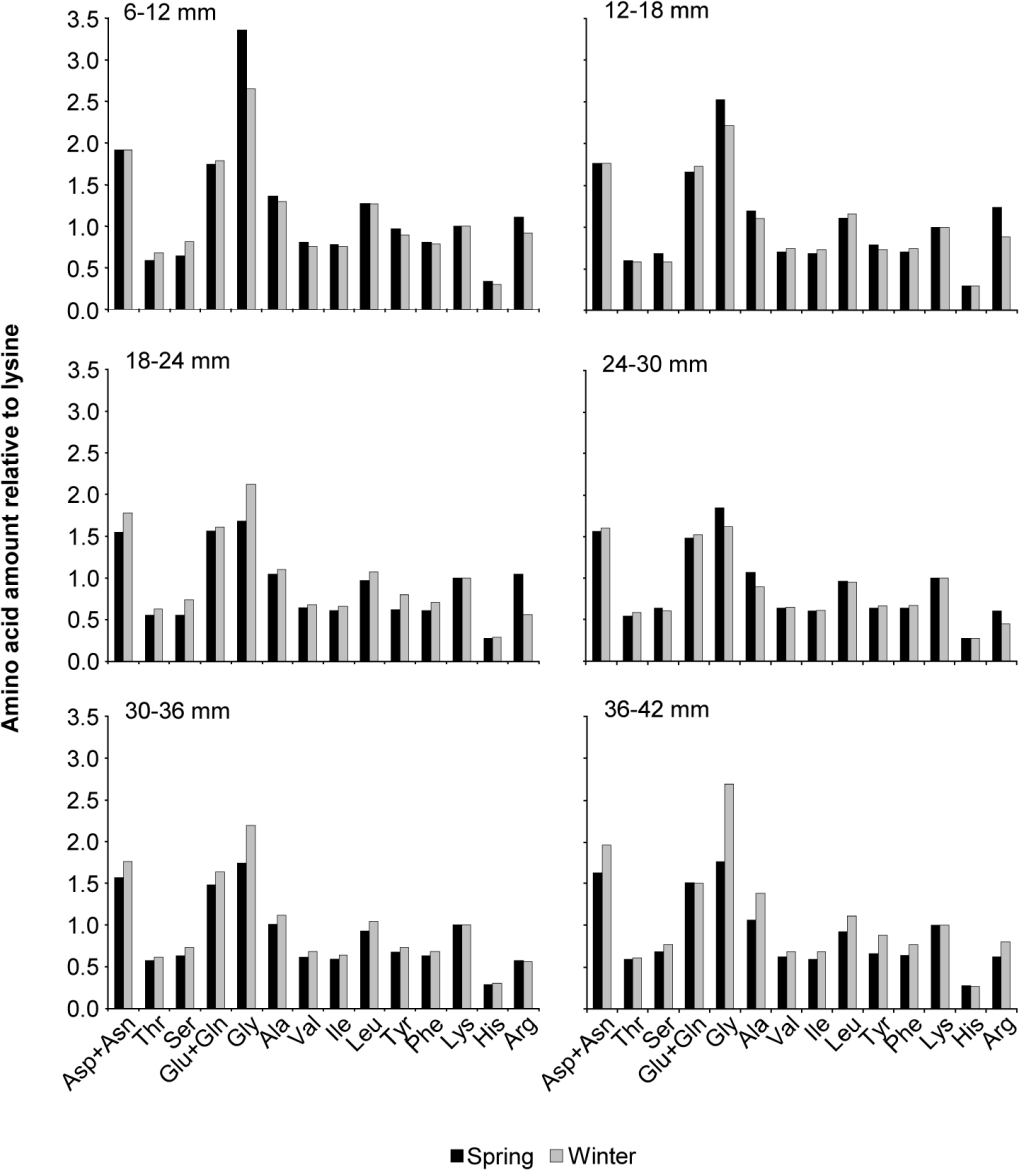
Amino acid amounts relative to lysine for six size classes of *Ischadium recurvum*. Amino acid amounts relative to Lysine for each size class (6–12, 12–18, 18–24, 24–30, 30–36, 36–42 mm) of *Ischadium recurvum* collected from the Chesapeake Bay in spring and winter 2007. Asp = Aspartic acid; Asn = Asparagine; Thr = Threonine; Ser = Serine; Glu = Glutamic acid; Gln = Glutamine; Gly = Glycine; Ala = Alanine; Val = Valine; Ile = Isoleucine; Leu = Leucine; Tyr = Tyrosine; Phe = Phenylalanine; Lys = Lysine; His = Histidine; and Arg = Arginine.

For convenience, the proportion of each amino acid was expressed relative to the amount of lysine (Figs 4 and 5). Lysine was chosen as the standard because it is particularly well studied and metabolically it is not used extensively for purposes other than protein synthesis. Aspartic acid and asparatine combined was higher in *M*. *lateralis* than *I*. *recurvum* as was arginine (Fig 4). *I*. *recurvum* contained slightly more glycine than *M*. *lateralis*. Seasonally smaller mussels in spring contained more glycine than smaller mussels in winter, however, in the larger mussels this relationship switched with more glycine available in the winter than spring (Fig 5). In the size class commonly consumed by scoters, 18–24 mm, the spring mussels contained more arginine relative to winter mussels. For the rest of the amino acid amounts the winter mussels contained more than the spring mussels.

### Shell Strength

**Fig 6:**
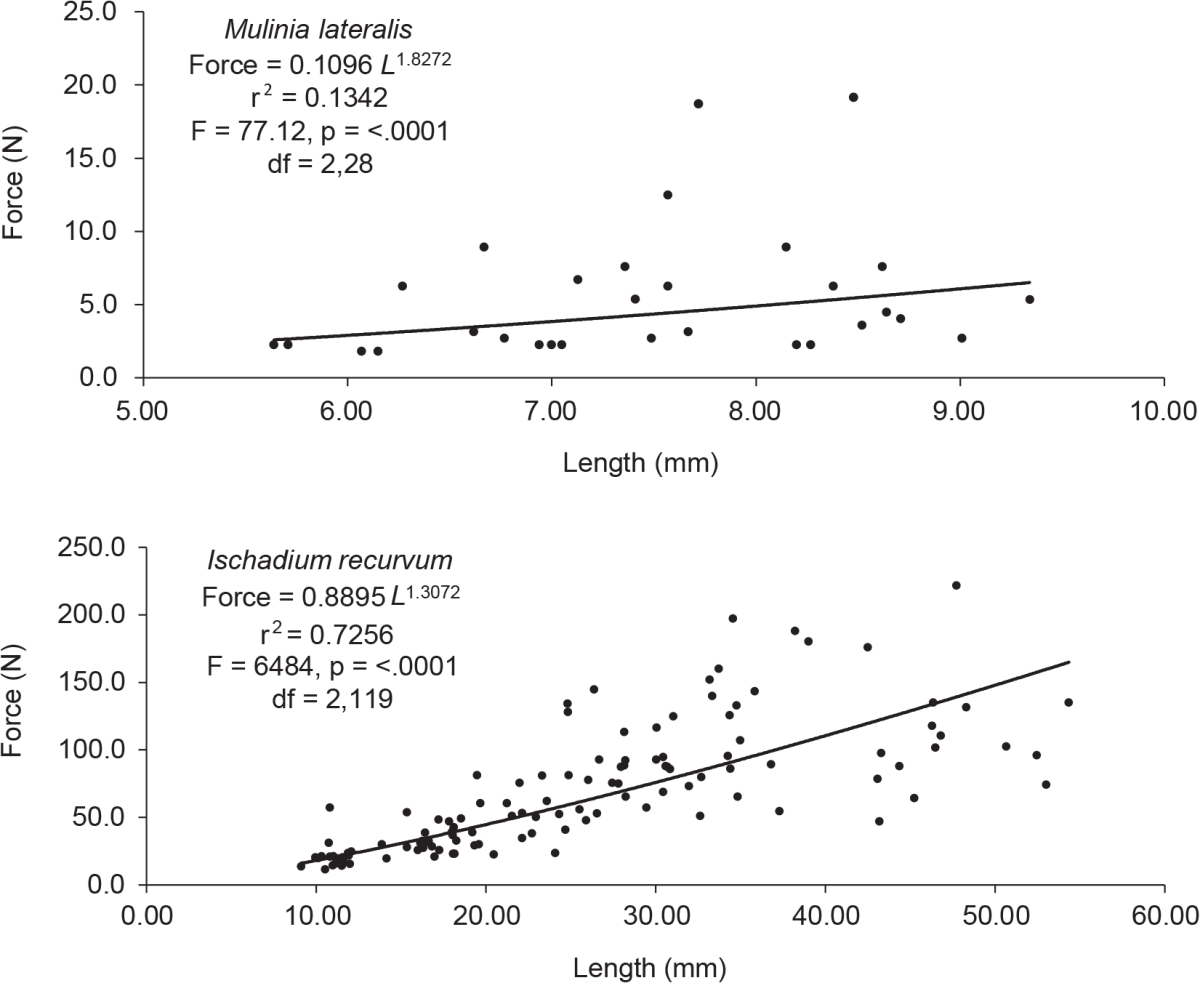
Shell strength relative to length of *Ischadium recurvum* and *Mulinia lateralis*. Shell strength, measured as the amount of force (N) needed to crack the shell, as a function of length (mm) for *Ischadium recurvum* and *Mulinia lateralis*, two common prey items consumed by wintering surf scoters on the Chesapeake Bay.

Shell strength of *I*. *recurvum* was significantly stronger than *M*. *lateralis* (F = 61.07, df = 1, 48, p < 0.0001). Shell strength increased nonlinearly with increasing length for both species (Fig 6).

### Metabolizable Energy

Scoters lost on average 5% of their body mass during the experiment (Table 4). There was no significant difference in initial body mass, final body mass, or mass loss between prey items (p = 0.8698, p = 0.8569, and p = 0.9584, respectively). Scoters were force fed 25g fresh mass of each prey item, but some regurgitated some of the prey. This occurred more frequently with the mussels than the clams, and, therefore, there was a significant difference in the amount of prey items digested (p = 0.0027). Ash, lipid, nitrogen, and gross energy were significantly different between the prey items (Table 4). There were no significant differences between prey items in the mass, ash, lipid, nitrogen, and gross energy in the excreta. There was no significant difference between the apparent digestible energy (ADE) of each prey item (p = 0.5733). There was no significant difference in the nitrogen energy balance (NEB) between prey species (p = 0.8110); in addition, apparent metabolizable energy (AME) was not significantly different between prey items (p = 0.3474).

**Table 4:** Means (± 1 SD) of surf scoter body mass before and after trials; of food (dry mass), ash (g DM), lipid (g DM), nitrogen (g DM), and gross energy (kJ) ingested; of guano (dry mass), ash (g DM), lipid (g DM), nitrogen (g DM), and gross energy (kJ) excreted; and of apparent digestible energy (ADE; %kJ), nitrogen energy balance (NEB; kJ), and apparent metabolizable energy (AME; %kJ) determined for surf scoters fed 25 g fresh mass (whole bivalves) of the hooked mussel (*Ischadium recurvum*) and dwarf surfclam (*Mulinia lateralis*), the top two prey items consumed by wintering surf scoters in the Chesapeake Bay.

		*I*. *recurvum*	*M*. *lateralis*	*p-value*
		n = 8	n = 8	
**Body Mass**	Initial Mass (g)	783 ± 97	775 ± 82	0.8698
	Final Mass (g)	705 ± 60	700 ± 48	0.8569
	Mass Loss (%)	5.0 ± 2.9	5.0 ± 2.4	0.9584
**Ingesta (DM)**	Food (g)	9.460 ± 2.780	13.85 ± 0.591	0.0027
	Ash (g)	7.930 ± 2.332	12.73 ± 0.543	< 0.0001
	Lipid (g)	0.058 ± 0.017	0.101 ± 0.004	< 0.0001
	Nitrogen (g)	0.010 ± 0.003	0.072 ± 0.003	< 0.0001
	Gross Energy (kJ) whole prey	47.33 ± 13.91	2.260 ± 0.096	< 0.0001
	Gross Energy (kJ) organic matter only	185.0 ± 54.38	147.4 ± 6.288	0.0075
**Excreta (DM)**	Guano (g)	8.598 ± 4.720	8.721 ± 5.502	0.9623
	Ash (g)	4.014 ± 3.020	3.964 ± 3.873	0.9772
	Lipid (g)	0.067 ± 0.035	0.074 ± 0.051	0.7294
	Nitrogen (g)	0.989 ± 0.418	0.987 ± 0.609	0.9952
	Gross Energy (kJ)	58.09 ± 26.09	58.13 ± 30.48	0.9978
**Assimilation**	ADE (%)	-34.14 ± 66.89	-193.1 ± 115.2	0.0218
	ADE (%)*	65.69 ± 17.11	59.82 ± 23.07	0.5733
	NEB (kJ)	-36.09 ± 15.25	-35.97 ± 22.22	0.9924
	AME (%)	48.90 ± 31.64	23.95 ± 35.30	0.2006
	AME (%)*	86.93 ± 8.09	84.78 ± 7.07	0.6169

* Based on energy value of organic matter only (no shell).

## Discussion

Larger mussels contain more energy per mussel than smaller ones, so one might expect the scoters to maximize the size of mussels ingested [26]. However, a number of studies have shown diving ducks selecting small or intermediate sizes of prey [22], [26], [27], [28], [29], [30], [31], [32]. In these studies, size selection has been explained by differential handling times, effects of meat:shell ratio on nutrient gain relative to passage rate, or as a means of avoiding risk of ingesting prey that are too large to swallow whole.

Stress tests on the dominant prey items showed *I*. *recurvum* had significantly harder shells than *M*. *lateralis*, probably because they are not buried in the sand to avoid predation as a clam would be. The mussels have adapted thicker shells and reside in large clumps as a way to reduce predation pressure from scoters and crabs. Seitz et al. [33] noted that epifaunal sessile prey are usually unable to evade predation and, therefore, must rely on armor, habitat complexity, residence in aggregations, and fast growth to a large size as techniques against predation. *I*. *recurvum* contained more energy and protein than *M*. *lateralis*; which should make it a more beneficial prey item. However, the increased ash content and harder shell should decrease the amount of energy that can be metabolized from it when compared to *M*. *lateralis*.

Hard-shelled prey, such as these bivalves, contain a high fraction of indigestible matter that can restrict available feeding time by limiting storage of food in the digestive tract [34], [35]. The meat of bivalves is highly digestible [36], however, their large bulk of calcium carbonate shell may limit nutrient assimilation by mechanically restricting access of digestive enzymes to the organic food component. In black ducks (*Anas rubripes*), Jorde and Owen [37] found higher digestibility for *Mytilus edulis* than for soft-shelled clams (*Mya arenaria*) when the ash content for *Mytilus* was approximately 12% lower than *Mya*. Richman and Lovvorn [26] reported that even though the ash content in *Potamocorbula amurensis* was 78–100% higher than *Macoma balthica*, the assimilation efficiency of *Potamocorbula* by lesser scaup (*Aythya affinis*) was 24% higher.

In our study, *I*. *recurvum* was 63% higher in ash than *M*. *lateralis*, which suggested that the digestibility of *I*. *recurvum* should be lower than *M*. *lateralis*. However, the digestibility of *I*. *recurvum* was 33% higher than *M*. *lateralis*. Karasov [24] hypothesized that most noncuticular protein and fat in arthropods can be digested and absorbed, as well as a fraction of the cuticle. If this were the case then it is possible that even with the higher ash content it could still be digested efficiently. In addition, the calcium carbonate in the shells can lower measurements of energy content in bomb calorimeters [38]. Therefore, the energy value for just organic matter (no shell) was also used to determine the amount of energy metabolized. The digestibility between the two prey items was not significantly different when based upon organic matter only energy values. Karasov [24] reported *MEC values for black African oystercatcher (*Haemoatopus moquini*) fed polychaetes (*Pseudonereis variegate*) and rock mussels (*Choromytilus meridionalis*) as 72%, black African oystercatcher fed limpit (*Patella granularis*) as 73%, lesser scaup (*Aythya affinis*) fed shrimp (*Gammarus* sp.) as 87%, and canvasback (*Aythya valisineria*) fed wild celery buds (*Vallisineria americana*) as 79%. Our results based on organic matter only energy values were in a similar range as the above reported values (87% *I*. *recurvum* and 83% *M*. *lateralis*). The high variability in these results could be resultant of the ducks being stressed when the feces were collected. This stress level could have enhanced their metabolism where they quickly utilized the energy from the prey and then were utilizing their endogenous reserves.

**Fig 7:**
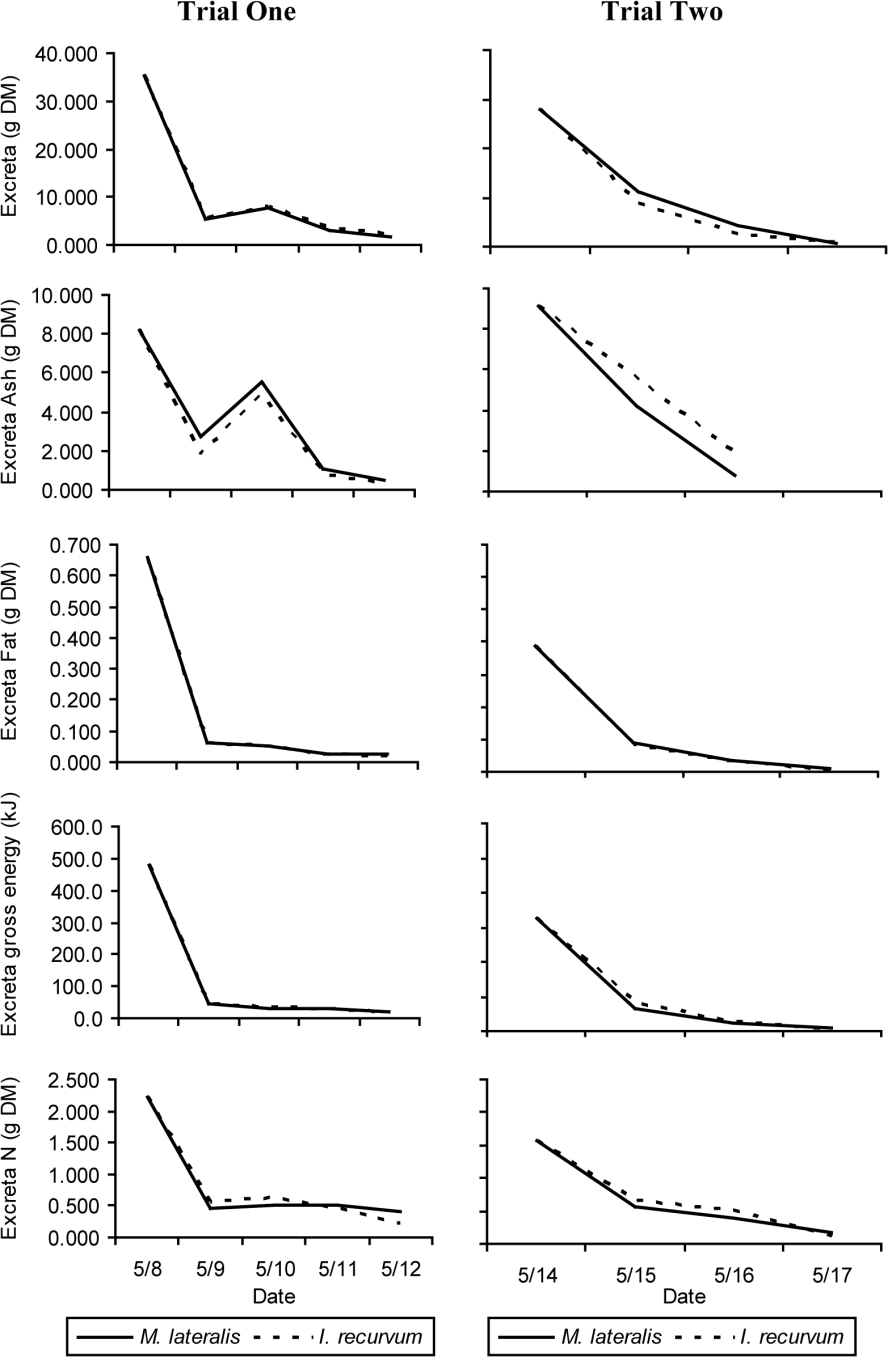
Excreta dry mass, ash, fat, nitrogen, and gross energy produced by surf scoters during foraging trials. The amount of excreta dry mass (g), ash (g DM), fat (g DM), nitrogen (g DM), and gross energy (kJ) produced each day during feeding trials on eight captive surf scoters fed *Mulinia lateralis* and *Ischadium recurvum*, the top two prey items obtained by wintering surf scoters in the Chesapeake Bay

Two assumptions made during the apparent metabolizable energy trials were that 1) there was no carry over of nutrients from their artificial maintenance diet and 2) that they excreted all the prey items in the 48 hr collection period. Grandy [39] reported that 95% of *Mytilus edulis* fed to black ducks was excreted after 50 minutes and Malone [40] reported that crayfish fed to mallards (*Anas platyrhynchos*) was 5% excreted in 66 minutes and 50% excreted in 86 minutes. To ensure that there was no carry over from the artificial diet the days the prey items were force fed (9 and 15 May) were excluded from analyses. Fig 7 plots excreta dry matter, ash, lipid, gross energy, and nitrogen by day. There was a spike in the ash content on 10 May probably due to shell being excreted by the scoters; however, this trend did not show up again on 16 May. The slight increase in lipid, nitrogen, and gross energy by the third day of collection could be due to an increase in endogenous losses, such as unrecovered digestive enzymes, mucus, and sloughed cells. Endogenous losses of amino acids could occur through loss of protein or nitrogen in the feces. This increase in endogenous losses could also be due to the fact that the ducks were force fed a hard-shelled prey item without becoming physiologically acclimated to digesting that prey item prior to the experiment. Karasov [24] noted that the digestive physiology of a bird can alter depending on the type of food source it was utilizing, such as switching from seeds to insects. In this study, it was decided that a 48 hour collection was sufficient for following reasons: 1) ducks in cage environment for a long time experience extreme stress so reaching steady state was not possible, 2) there were insufficient quantities of prey items to offer ducks food every day until they reached steady state or acclimate them to the prey item prior to the experiment, and 3) ducks could not be fasted for an extended period of time before as they would lose too much body weight and become ill. Future research should attempt to create a method that would allow for the measurement of metabolism without having to alter the behavior of ducks in such drastic measures.

### Summary

This study demonstrated that even though *I*. *recurvum* was higher in ash and contained a harder shell, this species contains more lipid, crude protein, and gross energy than *M*. *lateralis*. Despite the harder shell and higher ash content, *I*. *recurvum* was more efficiently digested than *M*. *lateralis*. Therefore, *I*. *recurvum* would be more advantageous as a prey item for surf scoters wintering in the Chesapeake Bay. However, alternative methodologies for assessing energy metabolized from these prey items are needed to verify these findings, especially if these methods are less stressful for the ducks. Ultimately, the foraging values of these prey items, the rate of intake of prey, and the relative expenditures must also be incorporated into a model to gain insight into the adaptive value of the prey items and feeding strategies for surf scoter.
